# Conformational diversity of collided ribosomes determines stress signaling

**DOI:** 10.64898/2026.06.16.730951

**Published:** 2026-09-10

**Authors:** Jeffrey J Li, Timo Denk, Matthias Thoms, Allen R Buskirk, Roland Beckmann, Rachel Green

## Abstract

Ribosome collisions trigger pathways that clear stalled ribosomes, and when sufficiently abundant, the integrated stress response (ISR) through GCN2 and the ribotoxic stress response (RSR) through ZAK. The inhibitors anisomycin (ANS), emetine (EME), and didemnin B (DDB) are commonly used to induce collisions in studying these responses. Here, we demonstrate that these drugs induce distinct signatures: ANS and DDB potently activate ZAK whereas EME does not. We define transcriptional programs induced by these inhibitors, where collisions induce the RSR and general inhibition of translation leads to an RSR-independent response. Surprisingly, we find that collisions induced by EME, unlike ANS, are not cleared by ASCC3. The cryo-EM structure of human disomes stalled by EME reveals its mechanism of inhibition and a conformation distinct from ANS-stalled disomes. These differences in collision geometry explain the different outcomes in quality control and signaling activation, showing how ribosome stalling events can yield distinct cellular responses.

## Full Text Availability

The license terms selected by the author(s) for this preprint version do not permit archiving in PMC. The full text is available from the preprint server.

